# Down-regulation of CHERP inhibits neuroblastoma cell proliferation and induces apoptosis through ER stress induction

**DOI:** 10.18632/oncotarget.20898

**Published:** 2017-09-15

**Authors:** Dunke Zhang, Feng Wang, Yi Pang, Xiao-xue Ke, Shunqin Zhu, Erhu Zhao, Kui Zhang, Lixue Chen, Hongjuan Cui

**Affiliations:** ^1^ State Key Laboratory of Silkworm Genome Biology, The Institute of Sericulture and Systems Biology, Southwest University, Chongqing, China; ^2^ Laboratory Research Center, The First Affiliated Hospital of Chongqing Medical University, Chongqing, China

**Keywords:** CHERP, cell proliferation, colony formation, neuroblastoma

## Abstract

Neuroblastoma is a childhood tumor that is derived from the sympathetic nervous system. In recent years, great progress has been made in our understanding of neuroblastoma. However, applying theories to improve disease outcomes remains challenging. In this study, we observed that calcium homeostasis endoplasmic reticulum protein (CHERP) was involved in the maintenance of neuroblastoma cell proliferation and tumorigenicity. Moreover, elevated CHERP expression was positively correlated with poor patient survival, whereas low CHERP expression was predictive of better outcomes. Additional functional studies showed that CHERP knockdown inhibited neuroblastoma cell proliferation *in vitro* and resulted in defective tumorigenicity *in vivo*. Moreover, CHERP depletion suppressed neuroblastoma cell proliferation by inducing endoplasmic reticulum stress and cell apoptosis. Considering the functional roles of CHERP in neuroblastoma development and maintenance, CHERP might function as a novel therapeutic target for neuroblastoma patients.

## INTRODUCTION

Neuroblastoma is a common and malignant tumor that develops from the sympathetic nervous system in children usually younger than 5 years of age [1, 2]. Neuroblastoma symptoms may include abdominal mass, pain, diarrhea, or a general feeling of discomfort. Neuroblastoma deaths account for 12% of cancer-associated deaths among children [3]. Despite great advances in clinical treatment in recent years, the 5-year overall survival of patients with malignant neuroblastoma remains lower than 40% [4]. Understanding the exact mechanism involved in the occurrence and development of neuroblastoma will help to improve preventive measures and treatment strategies for this poorly understood cancer. Here, we show that calcium homeostasis endoplasmic reticulum protein (CHERP) is involved in neuroblastoma cell proliferation, apoptosis and tumorigenicity. CHERP was first cloned and sequenced from a human erythroleukemia (HEL) cell expression library and is responsible for intracellular Ca^2+^ mobilization and cell growth [5]. Ryan et al. identified that CHERP interacts with RyR1 to control Ca^2+^ release from the endoplasmic reticulum (ER) and is primarily localized to the sarcoplasmic reticulum of rat soleus muscle [6]. Moreover, the study showed that CHERP co-localizes with IP3 receptors throughout the cytoplasmic and perinuclear regions in Jurkat T lymphocytes [7]. However, recent research has shown that CHERP interacts with 17S U2 small nuclear ribonucleoproteins, partially functions as a splicing factor and is present in nuclear speckles [8, 9]. Lin-Moshier et al. identified that CHERP is specifically located in the nucleus, including nuclear speckles [10]. Sasaki-Osugi et al. revealed that in HT1080 cells, CHERP participates in the regulation of alternative splicing of IP3R1 pre-mRNA [11]. To date, the role of CHERP in tumor biology remains unclear.

There are two primary pathways that regulate apoptosis in mammalian cells: the intrinsic and extrinsic pathways [12, 13]. In the intrinsic pathway, DNA damage, hypoxia, and growth factor deprivation activates the Bcl-2 family members (Bax and Bak) and results in the mitochondrial release of cytochrome c to induce apoptosis [14, 15]. The extrinsic pathway is activated by interaction of the tumor necrosis factor-related apoptosis-inducing ligand (TRAIL) with death receptors on the cell surface [16]. As previously reported, TRAIL-mediated apoptosis is a promising target for the clinical treatment of neuroblastoma [17–20]. Death receptor 5 (DR5) is an apoptosis-inducing receptor; once TRAIL binds to DR5, the adaptor Fas-associated DD (FADD) and pro-caspase-8 are recruited to the bound receptor. This interaction causes self-activation of pro-caspase-8, which processes downstream effector caspases such as caspase-3 and caspase-9 and thus leads to apoptosis [21–23]. Numerous lines of evidence have shown that ER stress (ERS) can up-regulate DR5 expression, and this process plays an important role in the initiation of apoptosis of human cancer cells [24, 25]. Additionally, activation of ER stress can inhibit the AKT/mTOR signaling pathway in numerous types of tumor cells [26–28]. Mammalian target of rapamycin (mTOR) is commonly activated in multiple tumors and forms two multiprotein complexes, mTORC1 and mTORC2, that control various cellular processes, including cell proliferation, metabolism, invasion, and autophagy [29–33]. mTORC1 can phosphorylate and inactivate the translational inhibitor 4E-BP to control protein synthesis and cell cycle processes [31].

In this study, we found that depletion of CHERP could induce ER stress, leading to activation of DR5 and inhibition of the AKT/mTOR signaling pathway. Additionally, treatment with the ER stress-specific inhibitor GSK2606414 partially rescued DR5-dependent apoptosis triggered by CHERP depletion. In brief, we reveal that the down-regulation of CHERP induces apoptosis in neuroblastoma cells by activating the ATF4/CHOP/DR5 signaling axis and inhibiting the AKT/mTOR signaling pathway. These results indicate that targeting CHERP might be a potential and novel therapeutic strategy for patients with neuroblastoma.

## RESULTS

### High CHERP expression in neuroblastoma patients is associated with poor prognosis

**Figure 1:**
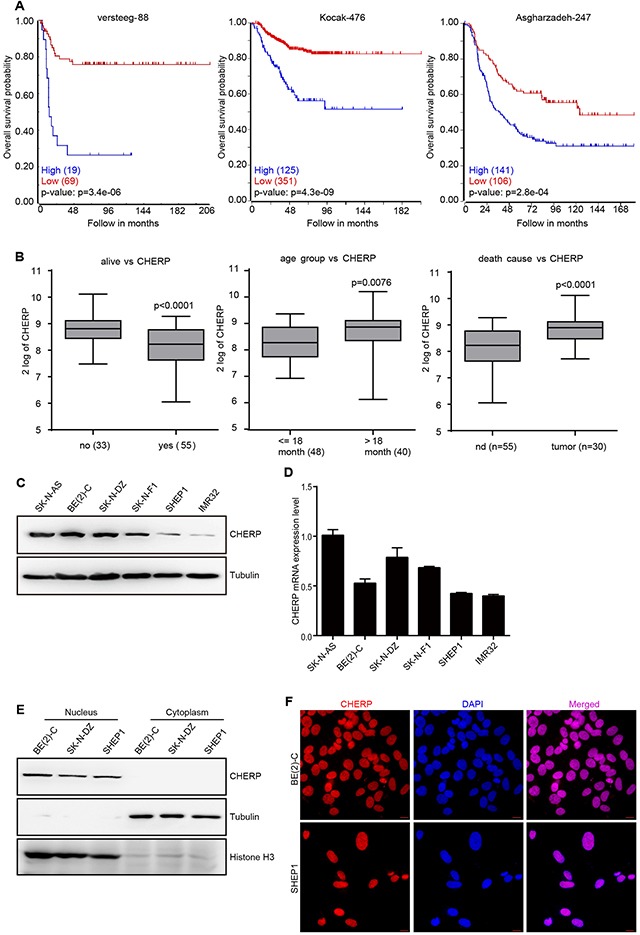
Elevated CHERP expression indicates a poor prognosis in neuroblastoma High CHERP expression is associated with poor outcomes in neuroblastoma patients. **(A)** Overall survival curve analysis of progression-free survival of the Neuroblastoma Prognosis Database for the Versteeg, Kocak and Asgharzadeh datasets; the P-values are indicated. **(B)** Box plot of CHERP expression levels in tumors from the dead and living groups, groups older than or younger than 18-months-old and the cause-of-death groups; the P-values are indicated. CHERP expression levels analyzed by western blotting **(C)** and qRT-PCR **(D)** in six neuroblastoma cell lines. **(E)** To analyze the localization of CHERP in BE(2)-C, SK-N-DZ and SHEP1 cells, nuclear and cytoplasmic extracts were prepared for western blotting. Tubulin and H3 histone were used as positive controls for nuclear and cytoplasmic proteins, respectively. **(F)** Immunostaining for CHERP (red) in BE(2)-C and SHEP1 cells; nuclei were stained with DAPI (blue). Scale bar for all microscopy images, 10 μm.

To investigate whether aberrant CHERP expression is associated with the prognosis of neuroblastoma patients, we used the Tumor Neuroblastoma public database (available from the online R2: Genomics Analysis and Visualization Platform). We selected three commonly used datasets (Versteeg, Kocak and Asgharzadeh), which contain data from 88, 476 and 247 neuroblastoma patients, respectively, to evaluate the effects of CHERP on overall patient survival. Kaplan–Meier analysis showed that high CHERP expression was associated with poor prognosis, whereas low CHERP expression was associated with good prognosis (Figure 1A). Moreover, based on the Versteeg dataset, CHERP was expressed significantly more highly in the overall death, older age (>18 month) and tumor-caused death groups than in the control group (Figure 1B). These results suggest that CHERP might be a diagnostic marker of neuroblastoma. To confirm this observation, we used data from the Neuroblastoma Prognosis Database, and the Kaplan–Meier analysis revealed that high CHERP expression was prognostic for poor outcomes in the Oberthuer Lab and Seeger dataset (Supplementary Figure 1A and 1B). Moreover, high CHERP expression significantly correlated with advancing tumor stage in the data extracted from the Kocak and Versteeg datasets (Supplementary Figure 1C). Furthermore, we analyzed whether CHERP expression levels were associated with MYCN expression in neuroblastoma patients. The results from the three datasets showed that CHERP expression increased significantly in the MYCN amplification group (Supplementary Figure 1D). In conclusion, higher CHERP expression is markedly associated with poor overall survival in neuroblastoma patients, which indicates that CHERP is a prognostic marker for neuroblastoma.

### CHERP is generally expressed in neuroblastoma and is located in nuclei

To confirm the CHERP expression levels in six neuroblastoma cell lines, we performed immunoblot analysis and qRT-PCR assays and observed that CHERP was commonly expressed in neuroblastoma cell lines with BE(2)-C cells exhibiting the highest levels and SHEP1 cells exhibiting the lowest levels (Figure 1C and 1D, respectively); thus, we chose these two cell lines for further studies. In rat soleus muscle tissue, CHERP was reported to co-localize with RyR1 in the sarcoplasmic reticulum [6]. However, recent data show that CHERP acts as a nucleoprotein and impacts cellular proliferation in HEK293 and SKBR3 cells [10]. To confirm the definitive localization of CHERP in neuroblastoma cells, we extracted nuclear and cytoplasmic proteins and used western blot assays to determine the precise location of CHERP. We found that in the neuroblastoma cell lines BE(2)-C, SK-N-DZ and SHEP1, endogenous CHERP was detected in the nucleus but not in the cytoplasm (Figure 1E). Moreover, immunofluorescence assays showed that CHERP localized to the nucleus in BE(2)-C and SHEP1 cells (Figure 1F). These observations demonstrate that CHERP is commonly expressed in neuroblastoma cells and is located in the nucleus.

### CHERP depletion inhibits neuroblastoma cell proliferation *in vitro*

**Figure 2:**
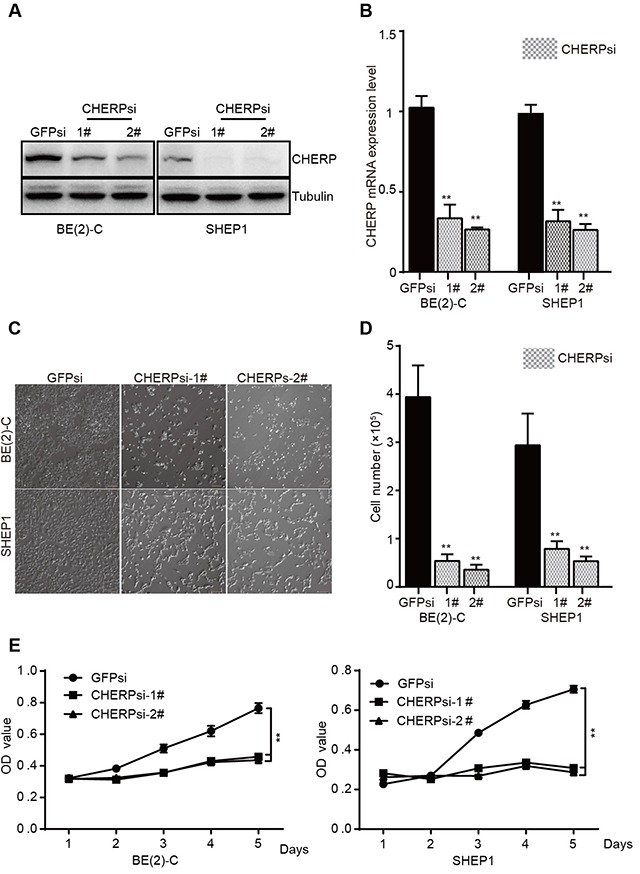
CHERP depletion decreases cell viability by inhibiting proliferation *in vitro* BE(2)-C and SHEP1 cells were transfected with CHERPsi-1# or CHERPsi-2# sequences with GFPsi as a biological control. The efficiency of shRNA-mediated interference was detected using western blotting **(A)** and qRT-PCR **(B)** in BE(2)-C and SHEP1 cells. **(C)** The number of cells decreased in CHERPsi-1# and CHERPsi-2# groups relative to that in the GFPsi group. Scale bar for all microscopy images, 100 μm. **(D)** BE(2)-C and SHEP1 cells expressing GFPsi, CHERPsi-1# or CHERPsi-2# were analyzed for cell counting 72 h after transfection. **(E)** CCK8 proliferation assays of BE(2)-C and SHEP1 cells expressing GFPsi, CHERPsi-1# or CHERPsi-2#. All data were analyzed using Student's t-test; *p < 0.05, **p < 0.001.

To verify the importance of CHERP in neuroblastoma, we employed a lentivirus system carrying small hairpin RNA (shRNA) to construct plasmids against target genes and then used these reconstructed lentiviruses (CHERPsi-1#, CHERPsi-2#, and GFPsi as a control) to infect BE(2)-C and SHEP1 cells. Immunoblot analysis and qRT-PCR assays showed that infecting cells with lentiviruses expressing targeted shRNAs resulted in significant CHERP down-regulation (Figure 2A and 2B), **P < 0.001. Cell number was dramatically lower in the CHERPsi group than in the control group (Figure 2C and 2D), **P < 0.001. This result was further supported by a growth curve assay, which revealed significant growth inhibition of cells subjected to CHERP down-regulation (Figure 2E). These data demonstrate that CHERP plays an indispensable role in neuroblastoma cell proliferation.

### CHERP depletion induces cell cycle arrest at G0/G1 phase in BE(2)-C and SHEP1 cells

**Figure 3:**
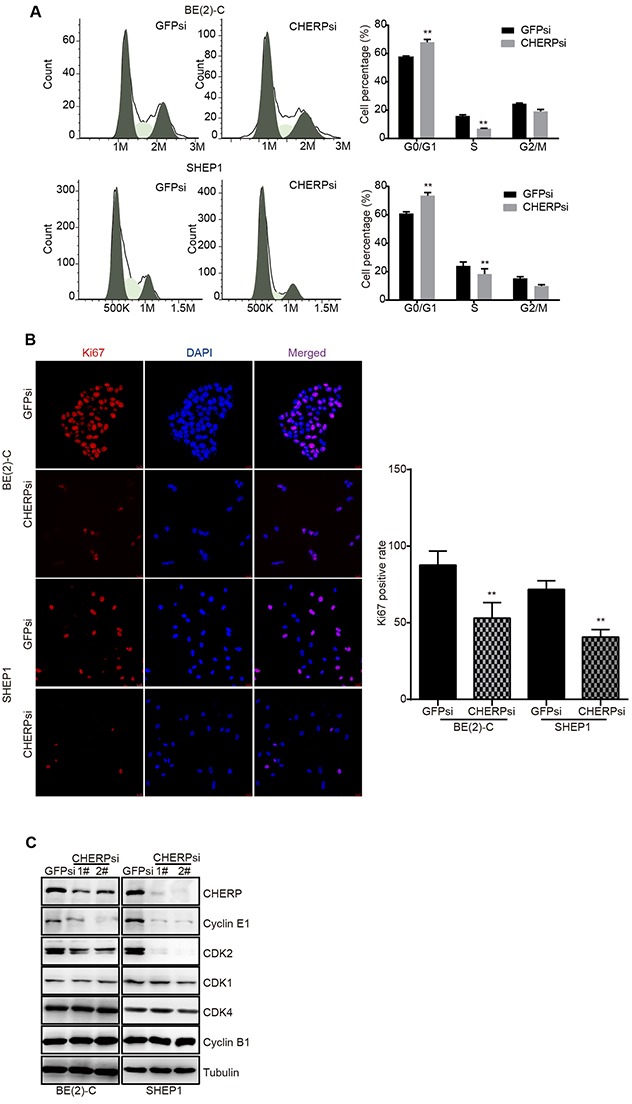
CHERP depletion induces cell cycle arrest *in vitro* CHERP depletion significantly increased the proportion of the cell population at G1 phase and decreased the proportion of cells in S phase in neuroblastoma. **(A)** Cell cycle analysis of BE(2)-C and SHEP1 cells using flow cytometry. **(B)** Ki67 staining analysis with Ki67 (red) and DAPI (blue). Quantification of Ki67-positive cells is presented on the right; all data were measured at least three times and were obtained from three independent images. Values are shown as the mean ± SD, **p < 0.001. **(C)** Expression of cyclins and CDKs associated with G1 phase as detected using western blotting.

The abovementioned data show that CHERP depletion inhibits neuroblastoma cell proliferation, and as reported, cell proliferation is often associated with cell cycle progression. Thus, we used flow cytometric analysis to further analyze the cell cycle status in BE(2)-C and SHEP1 cells with or without CHERP knockdown. Because both shRNAs (CHERPsi-1# and CHERPsi-2#) induced significant down-regulation of CHERP (Figure 2A and 2B), we used the CHERPsi-2# shRNA-expressing lentivirus for subsequent experiments. As shown in Figure 3A, cell cycle analysis of CHERP-depleted cells indicated a significant increase in the proportion of cells in the G0/G1 phase concomitant with a significant reduction in S phase compared with the corresponding proportions in the GFPsi cell population. These data showed that CHERP depletion induced cell cycle arrest at G0/G1 phase in BE(2)-C and SHEP1 cells, **P < 0.001. Furthermore, we stained cells for the proliferation marker Ki67 and observed that the Ki67-positive cell population was significantly decreased in CHERP-depleted cells relative to that in the control cells (Figure 3B), **P < 0.001. To validate these results, we evaluated G1/S checkpoint-associated proteins using western blot analysis. The expression levels of CDK2 and Cyclin E were decreased in CHERP-depleted cells, but there were no obvious changes in the expression levels of CDK1, CDK4 or Cyclin B1 (Figure 3C). These results suggest that CHERP represses neuroblastoma cell proliferation by reducing CDK2 and Cyclin E expression and inducing cell cycle arrest at G0/G1 phase.

### CHERP depletion induces cell apoptosis in BE(2)-C and SHEP1 cells

**Figure 4:**
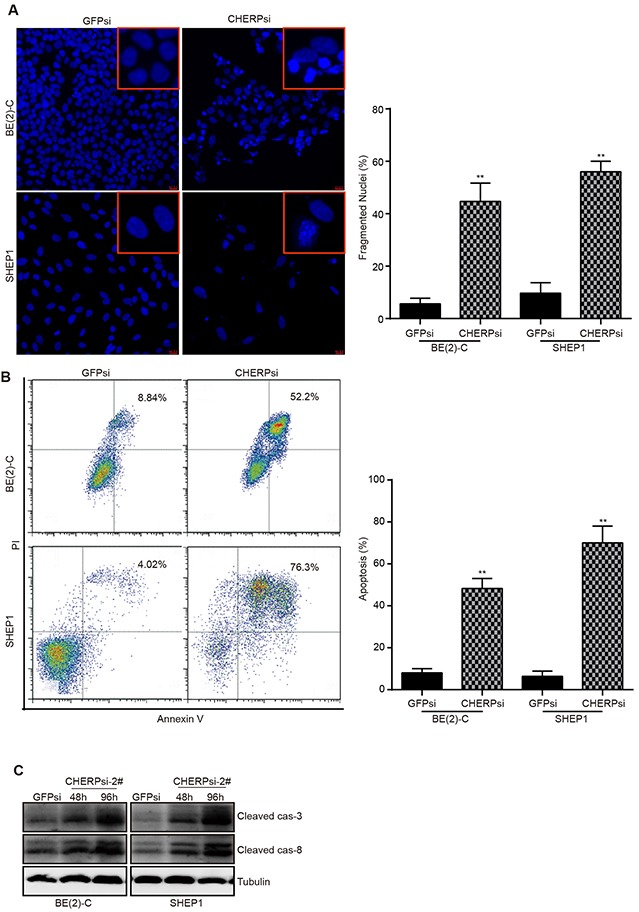
CHERP depletion induces neuroblastoma cells apoptosis **(A)** Hoechst 33258 staining for BE(2)-C and SHEP1 cells; regions of cells are magnified in the red frames. Scale bar for all microscopy images, 10 μm. Quantification of Hoechst 33258-stained cells is shown on the right; all data were measured at least three times and were obtained from three independent images. **(B)** Apoptosis analysis of BE(2)-C and SHEP1 cells using flow cytometry. Quantification of apoptotic cells is presented on the right, and all data were analyzed using Student's t-test, **P < 0.001. **(C)** Expression of apoptosis-associated proteins (cleaved caspase-3 and cleaved caspase-8) as detected using western blotting.

Because apoptosis may reduce cell proliferation and inhibit tumor growth, we detected nuclear condensation in CHERP-depleted cells using Hoechst 33258 staining. The nuclei of cells in the GFPsi group were large and round with a smooth nuclear membrane, whereas the nuclei in the CHERPsi group exhibited fragmentation, which indicated that CHERP depletion induced apoptosis and affected nuclear morphology (Figure 4A). As shown on the right, the quantitative analysis of cells with fragmented nuclei demonstrated that the proportion of cells with fragmented nuclei (%) in the CHERPsi group was dramatically higher than that in the GFPsi group, *P < 0.05. Furthermore, flow cytometry analysis of annexin-V-FITC and PI staining confirmed that the cell apoptosis rate was notably increased in the CHERP-depletion group (Figure 4B). Quantitative analyses of Figure 4B are shown on the right and illustrate that the cell apoptosis rate was dramatically increased by up to 52.2% in CHERPsi BE(2)-C cells and up to 76.3% in CHERPsi SHEP1 cells. Next, we analyzed the expression levels of several apoptosis-associated proteins using western blot analysis. Cleaved caspase-3 and cleaved caspase-8 were up-regulated in CHERP depletion cells compared with the levels in the GFPsi cells (Figure 4C). These results demonstrated that down-regulating CHERP could induce neuroblastoma cell apoptosis *in vitro*.

### CHERP depletion results in alterations in the drug sensitivity and cell viability of neuroblastoma cells in response to Dox

**Figure 5:**
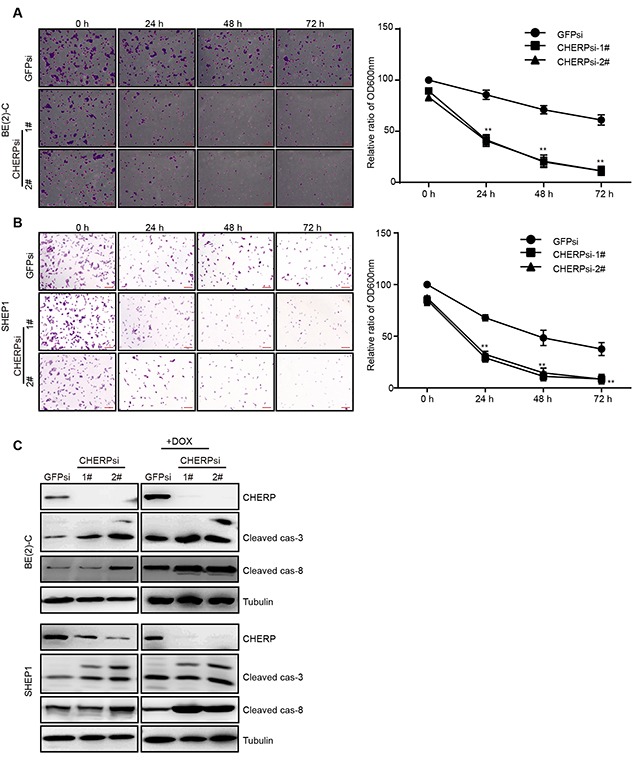
CHERP depletion results in alterations in the sensitivity of neuroblastoma cells to DOX Clonogenic assays of BE(2)-C **(A)** and SHEP1 **(B)** cells expressing GFPsi, CHERPsi-1# or CHERPsi-2# sequences and treated with 2 μM Dox for the indicated time (0 h, 24 h, 48 h or 72 h); colonies were stained with crystal violet solution and quantified. Scale bar for all microscopy images, 100 μm. Quantification of the stained colonies is shown in the right panel. All data are shown as the mean ± SD, **P ≤ 0.001. All p-values are based on analysis of control versus treatment. **(C)** Western blot analysis was performed to determine the expression of apoptosis-associated proteins (cleaved caspase-3 and cleaved caspase-8).

One of the important properties of cancer cells is their resistance to drugs. Therefore, we investigated whether depleting CHERP could affect the response of neuroblastoma to doxorubicin (Dox) treatment. We treated CHERP-depleted cells with 2 μM Dox for the indicated time. The clonogenic assays revealed that compared with the GFPsi group, the BE(2)-C (Figure 5A) and SHEP1 (Figure 5B) CHERPsi groups exhibited impaired Dox resistance in a time-dependent manner. The right panel is the quantitative analysis of the crystal violet staining after elution with 33% acetic acid and measurement at OD_600_. In addition, we treated CHERP-depleted cells with 2 μM Dox and extracted total protein for western blot assays. Consistent with the results from the clonogenic assays, the cleaved caspase-3 and cleaved caspase-8 protein levels showed dramatic increases compared with the levels in the control group (Figure 5C). These results showed that down-regulating CHERP could attenuate the drug sensitivity to Dox and cell viability of neuroblastoma cells.

### CHERP depletion inactivates mTOR and induces apoptosis in neuroblastoma cells by CHOP-dependent DR5 induction

**Figure 6:**
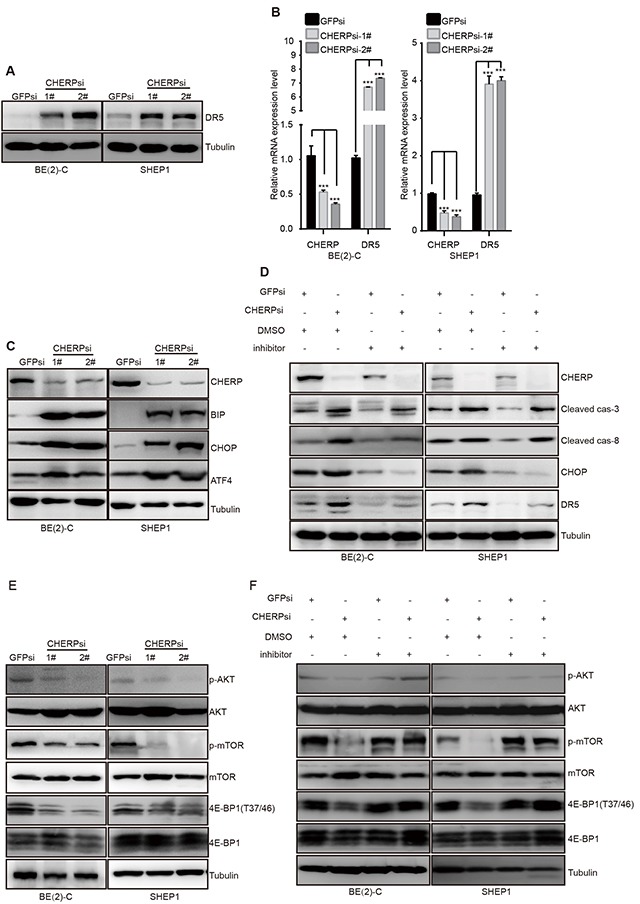
CHERP depletion induces neuroblastoma cell apoptosis via the ER stress pathway ER stress and AKT/mTOR signaling are involved in the induction of CHERP depletion-mediated neuroblastoma cell apoptosis. **(A)** Western blot analysis was performed to measure DR5 protein expression. **(B)** DR5 mRNA levels as evaluated by qRT-PCR; the values represent the mean ± S.D. (n = 3). All data were analyzed by Student's t-test, **P< 0.001. **(C)** ER stress-associated proteins as analyzed by western blotting. BE(2)-C and SHEP1 cells expressing GFPsi, CHERPsi-1# or CHERPsi-2# sequences were treated with 50 μg of the ERS inhibitor GSK2606414, and the indicated proteins were detected using western blotting **(D)**. AKT, mTOR and 4E-BP1 expression levels were also analyzed using western blotting **(E and F)**.

Thus far, we have shown that CHERP depletion can induce neuroblastoma cell apoptosis and increase the expression of cleaved caspase-3 and cleaved caspase-8 expression. Although most human neuroblastoma cells exhibit drug resistance because of their low levels of caspase-8 expression, previous reports have shown that TRAIL-receptor 2 (TRAIL-R2/DR5/TNFRSF10b) is an important upstream effector of caspase-8 [34]. Thus, we then examined the DR5 levels in CHERP-depleted cells. Consistent with our expectations, DR5 was significantly up-regulated at both the mRNA and protein levels (Figure 6A and 6B). As previously reported, ER stress in response to CHOP can induce DR5 transcription n human carcinoma cells [35]. To determine whether DR5 transcription is regulated by ER stress in neuroblastoma cells, we examined the ER stress marker proteins BIP, ATF4 and CHOP. Depletion of CHERP in BE(2)-C and SHEP1 cells dramatically increased BIP, ATF4 and CHOP expression, which suggested that ER stress was involved in neuroblastoma cell apoptosis induced by CHERP depletion. To confirm this observation, we treated cells with the ER stress inhibitor GSK2606414 [36, 37]. The addition of 50 μg of GSK2606414 resulted in a reduction in the cleaved caspase-3 and cleaved caspase-8 expression levels to those observed in the GSK2606414-negative group. CHOP and DR5 expression also decreased in CHERP-depleted BE(2)-C and SHEP1 cells treated with GSK2606414 (Figure 6D). As previously reported, the activation of ERS promoted apoptosis and reversed chemoresistance in human small cell lung cancer (SCLC) cells by inhibiting the PI3K/AKT/mTOR pathway [26]. As shown in Figure 6E, we found that the levels of AKT, mTOR and 4E-BP1 phosphorylation were attenuated after CHERP depletion, which indicated that the AKT/mTOR/4E-BP1 pathway was involved in neuroblastoma cell apoptosis mediated by CHERP depletion. Furthermore, after GSK2606414 treatment, we found that AKT, mTOR and 4E-BP1 phosphorylation were rescued; these results suggested that CHERP depletion could inhibit the AKT/mTOR/4E-BP1 pathway via activation of ERS (Figure 6F). In conclusion, these results indicate that ER stress is involved in neuroblastoma cell apoptosis induced by CHERP depletion; moreover, up-regulation of DR5 and inhibition of the AKT/mTOR/4E-BP1 pathway represent two separate downstream events of this process.

### CHERP depletion reduces neuroblastoma cell colony formation and represses tumorigenicity *in vitro* and *in vivo*

**Figure 7:**
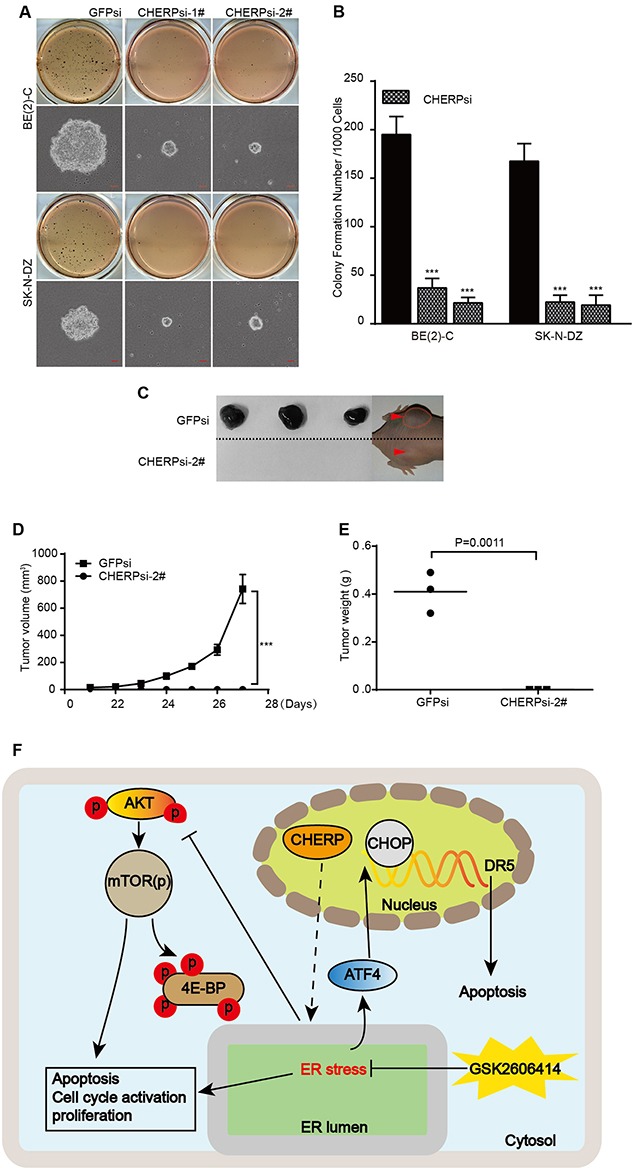
CHERP depletion impairs colony growth and tumorigenicity of neuroblastoma cell lines *in vitro* and *in vivo* **(A)** Soft agar colony-formation assays with BE(2)-C and SK-N-DZ cells expressing GFPsi, CHERPsi-1# or CHERPsi-2# sequences. Colonies were stained with MTT and scored, and the quantification of the stained colonies is shown in **(B)**. Scale bars in all microscopy images, 50 μm. **(C)** Representative tumors from the GFPsi and CHERPsi-2# groups. Mice were imaged for 1 week after the tumors became palpable. **(D)** Tumor growth curve of the GFPsi and CHERPsi-2# cells injected into immunodeficient mice. **(E)** The weights of the xenograft tumors formed by the GFPsi and CHERPsi-2# BE(2)-C cells are indicated along with their p-values. All values represent the mean ± S.D. (n = 3). Student's two-tailed t-test, **P < 0.001. **(F)** Diagram of the mechanism by which CHERP regulates neuroblastoma cell proliferation and apoptosis.

To investigate whether CHERP depletion could affect tumorigenicity in neuroblastoma cells, we performed a soft-agar colony-formation assay. The size and number of colonies were obviously reduced in CHERP-depleted cells, **P < 0.001 (Figure 7A and 7B). Furthermore, we investigated the role of CHERP in the tumorigenicity of neuroblastoma cells *in vivo* by subcutaneously implanting CHERP-depleted BE(2)-C cells into immunodeficient mice. In the animal model images, red arrows and dashed lines indicate the positions of the xenograft tumors. CHERP depletion impaired tumor growth compared with that in the GFPsi group, and mice injected with CHERPsi-2# cells did not form xenograft tumors **P < 0.001 (Figure 7C–7E). Taken together, these experiments indicate that CHERP plays a key role in the colony-forming ability and tumorigenicity of neuroblastoma. Figure 7F presents a diagram of the mechanism by which CHERP regulates neuroblastoma cell proliferation and apoptosis. In brief, CHERP depletion induces ER stress and CHOP-dependent DR5 transcription, attenuates mTOR and 4EBP1 phosphorylation, and ultimately induces neuroblastoma cell apoptosis.

## DISCUSSION

Although some studies performed in mammalian cells have elucidated some functions of CHERP, such as controlling intracellular Ca^2+^ mobilization and cell growth, acting as part of splicing factors and participating in post-transcriptional regulation of splicing variants in Ca(2+) signaling pathways, etc. [5, 10, 11], there has been little progress in understanding the role of CHERP in humans, especially in human cancer cells. Here, we determined that CHERP expression was a potential prognostic marker in neuroblastoma patients. High CHERP expression was associated with poor outcomes, and low expression was associated with a good prognosis. Moreover, CHERP expression was associated with patient age, cause of death and stages and MYCN status of neuroblastoma patients. O’Rourke et al. cloned CHERP and found that this protein is localized to the ER [5, 7]. Ryan et al. showed that CHERP interacts with RyR1 in the sarcoplasmic reticulum of rat soleus muscle [6]. Lin-Moshier et al. and Sasaki-Osugi et al. found that CHERP acts as a nuclear protein [10, 11]. To date, the definitive cellular location of CHERP has not been established, which may indicate multiple functions of CHERP in different cells or locations. Here, we verified that CHERP is located in the nuclei in neuroblastoma cells and revealed its role in controlling cell proliferation and apoptosis during neuroblastoma initiation and development. Moreover, CHERP depletion combined with Dox treatment confirmed that CHERP plays an important role in the survival and drug resistance of neuroblastoma cells. Advances in genetics have resulted in remarkable progress in neuroblastoma treatment over the years. Our discovery of the functions of CHERP in neuroblastoma cell proliferation, apoptosis and tumorigenicity combined with integrated drug treatments may provide a new potential strategy for clinical treatment of neuroblastoma.

Further mechanistic studies revealed that CHERP depletion induces ERS and CHOP-dependent DR5 transcription; attenuates AKT, mTOR and 4EBP1 phosphorylation; and ultimately promotes neuroblastoma cell apoptosis. Emerging evidence indicates that targeting CHOP/DR5 mediates apoptosis in cancer. mTOR has been well established as a master regulator of cell growth, motility, survival, protein synthesis, and transcription. Over-activation of mTOR leads to considerable accumulation of unfolded protein in the ER and triggers ERS [38, 39]. In addition, activation of ERS can inhibit the AKT/mTOR signaling pathway and promote cell autophagy and apoptosis in numerous tumors [40–42]. Our findings reveal that CHERP depletion can induce ERS, although the precise mechanism of this process requires further exploration.

Furthermore, our *in vitro* and *in vivo* experimental results demonstrate that the status of CHERP is essential for clone forming ability and tumorigenicity of neuroblastoma cells. Neuroblastoma cells with depleted CHERP minimally form xenotransplanted tumors in nude mice. We speculated that CHERP depletion leads to ERS and inhibits neuroblastoma initiation and that tumor progression is related to the subcellular localization of CHERP in cells. Our results validated previous reports that CHERP is located in the nucleus and regulates the function of the U2 snRNA spliceosomal complex [43]; therefore, we speculated that depletion of CHERP leads to U2 snRNA spliceosomal complex dysfunction and subsequently triggers ERS, interferes with cell proliferation, induces cell apoptosis and inhibits tumorigenicity. These results suggest that CHERP plays a crucial role in tumor formation and development, and this role should be studied further to fully explain the function of CHERP in neuroblastoma initiation and progression.

In summary, our study identifies CHERP as a potential target for the treatment of neuroblastoma and reveals the following novel findings: (a) CHERP is generally expressed in neuroblastoma, and high CHERP expression is associated with poor prognosis in neuroblastoma patients; (b) CHERP depletion inhibits neuroblastoma cell proliferation and induces cell cycle arrest at G0/G1 phase; (c) CHERP depletion triggers ERS and induces cell apoptosis via CHOP-dependent DR5 induction and inhibition of the AKT/mTOR signaling pathway in neuroblastoma cells; and (d) CHERP depletion impairs the clone-forming ability and tumorigenicity of neuroblastoma cells. Overall, our findings provide new insights into the mechanism whereby CHERP regulates neuroblastoma cell fate and provides a potential therapeutic target for neuroblastoma.

## MATERIALS AND METHODS

### Cells and cell culture

The human neuroblastoma cell lines BE(2)-C, SK-N-DZ, SK-N-F1, SHEP1 and IMR32 were purchased from ATCC (Rockville, MD, USA), and all cell lines except BE(2)-C were cultured in complete medium containing DMEM (Life Technologies, Grand Island, NY, USA) supplemented with 10% fetal bovine serum (Invitrogen) and 1% penicillin/streptomycin (Life Technologies, Grand Island, NY, USA). BE(2)-C cells were cultured in DMEM/F-12 complete medium. All cells were maintained at 37°C in a humidified incubator containing 5% CO_2_.

### Drug treatment

Doxorubicin (Dox) was purchased from Abcam (ab120629) and dissolved in double-distilled water. Cells infected with targeted lentiviruses for 48 h were grown in the presence or absence of 2 μM Dox for 24 h, 48 h and 72 h. At the indicated times, cells were stained with crystal violet. The stained cells were routinely examined using an inverted microscope, and the absorbance was measured at a wavelength of 600 nm. Cells treated with 2 μM Dox at 48 h were collected for further western blot analysis. For the GSK2606414 (Selleckchem, S7307) treatment assay, cells were cultured in the presence or absence of 50 μg GSK2606414 for 2 h, after which they were infected with targeted lentiviruses for 48 h. Cells were collected for further western blot analysis.

### Transfection and viral infection

The pLKO.1 vector was combined with 0.5 μg of pLP1, pLP2 and pLP/VSVG plasmids and transfected into 293FT cells with Lipofectamine 2000 transfection agent, and the cells were cultured for 48 h. The supernatant was collected and filtered through a 0.45-μm filter. These virus-containing supernatants were used to infect target cells for 24 h, and the remaining lentiviruses were stored at -80°C. After two rounds of infection, the infected cells were cultured with 2 mg/ml puromycin for one day to establish a stable cell line.

### Cell growth assay

The cell growth curve was produced using a CCK-8 (Beyotime) assay. After cells were infected, they were seeded into 96-well plates at 800 cells per well and cultured overnight. Then, 10 μl of CCK-8 was added to each well and incubated at 37°C for 2 h, and the absorbance was measured at a wavelength of 450 nm.

### Patient data analysis

Patient data were analyzed as previously described. Briefly, the overall patient survival plots and gene expression datasets were analyzed using the R2: Microarray Analysis and Visualization Platform (http://hgserver1.amc.nl/cgi-bin/r2/main.cgi) and the Oncogenomics Section Data Center (http://pob.abcc.ncifcrf.gov/cgi-bin/JK) [44]. The survival curves and P-values (log-rank test) were downloaded online, and the gene expression data were obtained from datasets and analyzed using GraphPad Prism (version 6.0). All cutoff values for separating the high- and low-expression groups were determined using either the online R2 or Oncogenomics database algorithms [45].

### Western blot analysis

Cells were lysed in RIPA Lysis Buffer (Beyotime) and mixed with PMSF (Beyotime), and protein concentrations of the resulting lysates were quantified using a BCA Protein Assay Kit (Beyotime). A total of 30 μg of protein from each sample was separated using SDS-PAGE on a 10% gel and transferred to a polyvinylidene fluoride membrane. The membranes were probed with the following antibodies: 1:1000 rabbit polyclonal anti-CHERP antibody (ab15951, Abcam); 1:1000 rabbit polyclonal anti-cleaved caspase-3 antibody (AC033, Beyotime); mTOR substrate antibodies (9862, CST); 1:800 polyclonal anti-DR5/TNFRSF10B antibody (BS60081, Bioworld); 1:1000 polyclonal anti-Bip antibody (3177, CST); 1:1000 CHOP polyclonal antibody (2895, CST); 1:500 AKT polyclonal antibody (4691, CST); 1:1000 polyclonal anti-P-Akt (Ser473) antibody (4060, CST); 1:800 polyclonal anti-cleaved caspase-8 antibody (E1A5267, EnoGene); and 1:800 polyclonal anti-ATF4 antibody (E2A6008, EnoGene). For the cell cycle protein assay, either a cell cycle regulation antibody sampler kit (9932, CST) or a 1:2000 α-tubulin mouse polyclonal antibody (AT819, Beyotime) was used. The secondary antibodies utilized were horseradish peroxidase-conjugated goat anti-mouse (A0216, Beyotime) and goat anti-rabbit IgG (A0208, Beyotime).

### Immunofluorescence

For immunofluorescence staining, 2 × 10^5^ cells were grown in a 24-well plate and fixed with 4% paraformaldehyde at room temperature for 20 min. After the cells were permeabilized with 0.3% Triton X-100 for 10 min and blocked with 5% goat serum for 30 min in a 37°C incubator, primary antibody was added to the cells and incubated overnight at 4°C. The following day, the cells were incubated with the appropriate secondary antibodies at 37°C for 40 min, and 4,6-diamidino-2-phenylindol (DAPI) was added to stain the nuclei. To detect CHERP, an antibody was purchased from Abcam and used at a dilution of 1:200. For Ki67 (CST, 9449) immunofluorescence staining, we used a mouse monoclonal antibody at a dilution of 1:800. Antibodies conjugated to either Alexa Fluor 488 (Invitrogen, A21206) or Alexa Fluor 594 (H+L) (Invitrogen, A21203) were used at a dilution of 1:2000.

### RNA purification and RT-PCR

Total RNA was purified using TRIzol reagent (Takara) according to the manufacturer's instructions, and 2 μg of RNA was reverse transcribed (RT) to obtain cDNA. qRT-PCR was then performed using SYBR® Green PCR Master mix (TaKaRa), and the reactions were performed in triplicate using a StepOnePlus 7500 real-time PCR system (Applied Biosystems). GAPDH, an internal control, was used to normalize mRNA expression.

### Apoptosis analysis

After cells were infected, they were stained with Hoechst 33258 (Beyotime), imaged with a fluorescence microscope (OLYMPUS) and scored. In preparation for the flow cytometry analysis, the infected cells were harvested and stained with propidium iodide and fluorescein isothiocyanate (FITC)-labeled annexin V for 30 min at room temperature. The stained cells were then counted and analyzed using a flow cytometer (BD).

### Soft-agar assays

After cells were infected, 1500 cells in 1.5 ml of 0.3% Noble agar containing supplemented media (10% FBS + DMEM) were implanted in 6-well plates containing 1 ml of 0.6% Noble agar and supplemented media mixture. Images were viewed using a microscope 3 weeks after the initial seeding. Colonies were stained with MTT, and the stained colonies were scanned and scored by a scanner (Lenovo).

### Xenograft assay

All animal studies were performed at Southwestern University (SWU) according to the Purdue Animal Care and Use Committee. BE(2)-C cells were transfected with GFPsi and CHERPsi and expanded in DMEM/F12 supplemented with 10% FBS. Both flanks of 4-week-old NOD/SCID female mice (Beijing Laboratory Animal Research Center, China) were subcutaneously injected with 2 × 10^6^ infected cells in 200 μl of PBS. After seven days, tumor growth was measured using a Vernier caliper, and tumor volume was calculated using the formula 4/3πr^3^. At 14 days after tumor injection, the tumors were excised and weighed.

All data were confirmed by at least three independent experiments, and the results are presented as the mean ± S.D. P-values were calculated via Student's t-test using GraphPad Prism (version 6.0), and P < 0.05 was considered significant.

## SUPPLEMENTARY MATERIALS FIGURES AND TABLES



## Abbreviations

CHERP: calcium homeostasis endoplasmic reticulum protein
ERS: ER stress
Dox: doxorubicin
mTOR: mammalian target of rapamycin
4E-BP1: eukaryotic initiation factor 4E-binding protein-1
