# Supplementary material for: Down-regulation of CHERP inhibits neuroblastoma cell proliferation and induces apoptosis through ER stress induction
Source: Oncotarget. 2017 Sep 15;8(46):80956–70. doi: 10.18632/oncotarget.20898 (PMC5655253; doi:10.18632/oncotarget.20898)
Supplement: Supplementary file 1 [file oncotarget-08-80956-s001.pdf]

Down-regulation of CHERP inhibits neuroblastoma cell proliferation and induces apoptosis through ER stress induction

SUPPLEMENTARY MATERIALS

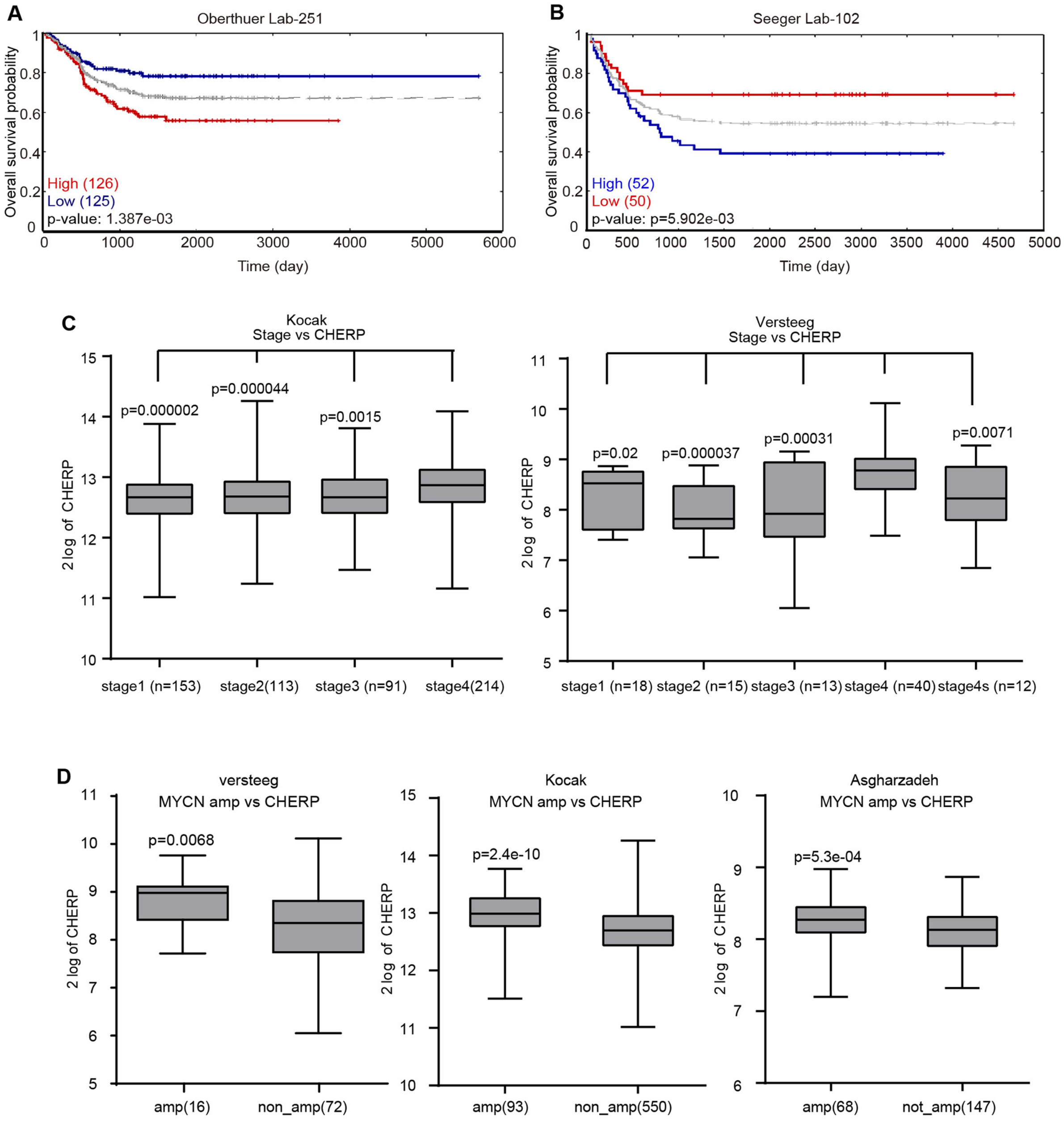

Supplementary Figure 1: High CHERP expression is associated with poor outcome in neuroblastoma patients. Overall survival curve analysis of progression-free survival for the Oberthuer Lab **(A)** and Seeger Lab **(B)** data sets in the Neuroblastoma Prognosis Database; the P-values are indicated. **(C)** Box plot of CHERP expression levels in stage (ST) 1–4 tumors (ST1 vs. ST4, ST2 vs. ST4, ST3 vs. ST4, or ST4 vs. ST4s) in the Kocak and Versteeg data sets; the P-values are indicated. **(D)** Box plot of CHERP expression levels in tumors from MYCN amplification and non-amplification groups in Versteeg, Kocak and Asgharzadeh databases; the P-values are indicated.
